# Supplementary material for: Unraveling Complex Hysteresis Phenomenon in 1,2-Dipalmitoyl-sn-Glycero-3-Phosphocholine Monolayer: Insight into Factors Influencing Surface Dynamics
Source: Int J Mol Sci. 2023 Nov 13;24(22):16252. doi: 10.3390/ijms242216252 (PMC10671618; doi:10.3390/ijms242216252)
Supplement: Supplementary file 1 [file ijms-24-16252-s001.zip › ijms-2701345-supplementary.pdf]

Unraveling Complex Hysteresis Phenomenon in 1,2-Dipalmitoyl-sn-Glycero-3-Phosphocholine Monolayer: Insight into Factors Influencing Surface Dynamics

Wisnu Arfian A. Sudjarwo * and José L. Toca-Herrera *

Institute of Biophysics, Department of Bionanosciences, University of Natural Resources and Life Sciences Vienna (BOKU), 1190 Vienna, Austria

* Correspondence: Correspondence: wisnu.sudjarwo@boku.ac.at (W.A.A.S.); jose.toca-herrera@boku.ac.at (J.L.T.-H.)

SUPPLEMENTARY MATERIAL

Table S1. Values of hysteresis energy for low and high expansion rates.

Compression rates (mm/min)	Hysteresis energy (J) (constant expansion rate 5 mm/min)	Hysteresis energy (J) (constant expansion rate 25 mm/min)
5	$6.58*10^{-22} \pm 8.28*10^{-23}$	$5.11*10^{-22} \pm 1.59*10^{-23}$
10	$6.18*10^{-22} \pm 1.01*10^{-22}$	$4.26*10^{-22} \pm 6.53*10^{-23}$
15	$6.24*10^{-22} \pm 1.06*10^{-22}$	$2.75*10^{-22} \pm 4.87*10^{-23}$
20	$5.56*10^{-22} \pm 1.37*10^{-22}$	$2.32*10^{-22} \pm 3.59*10^{-23}$
25	$5.49*10^{-22} \pm 1.36*10^{-22}$	$1.94*10^{-22} \pm 1.33*10^{-23}$

Equation	y = a + b*x	
Plot	Expansion 5mm/min	Expansion 25 mm/min
Weight	Instrumental	
Intercept	6.84534E-22 ± 1.58941E-23	5.85378E-22 ± 2.12375E-23
Slope	-5.48517E-24 ± 1.101E-24	-1.5894E-23 ± 1.1091E-24
Residual Sum of Squares	0.07203	3.50214
Pearson's r	-0.94454	-0.99277
R-Square (COD)	0.89217	0.9856
Adj. R-Square	0.85622	0.9808

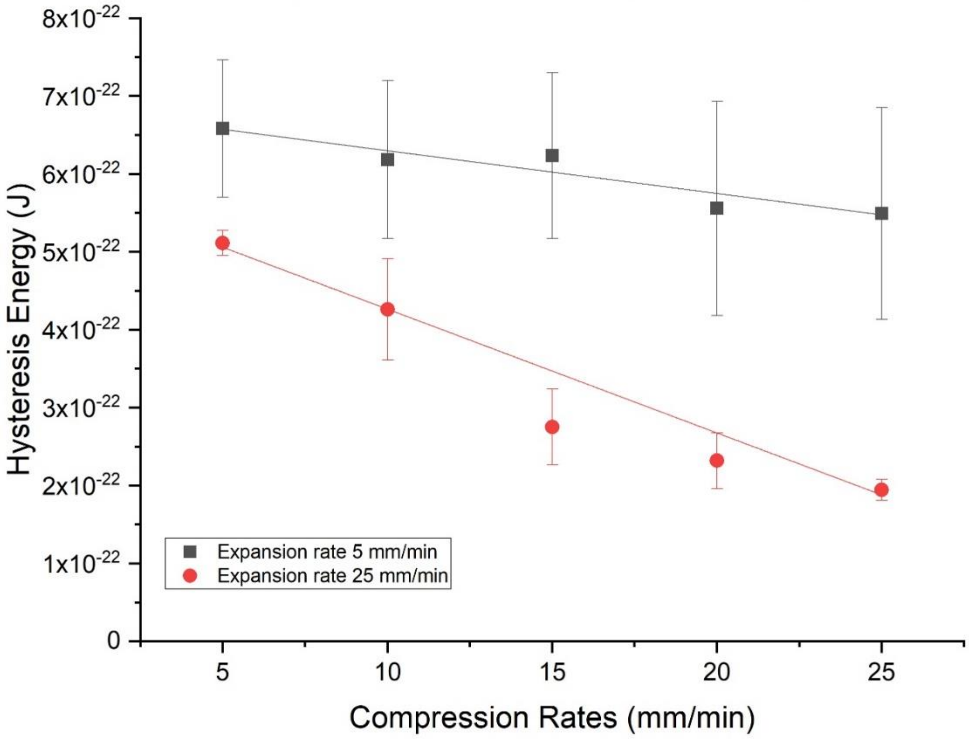

Figure S1. Linear regression of hysteresis energy as a function of compression and expansion rates.

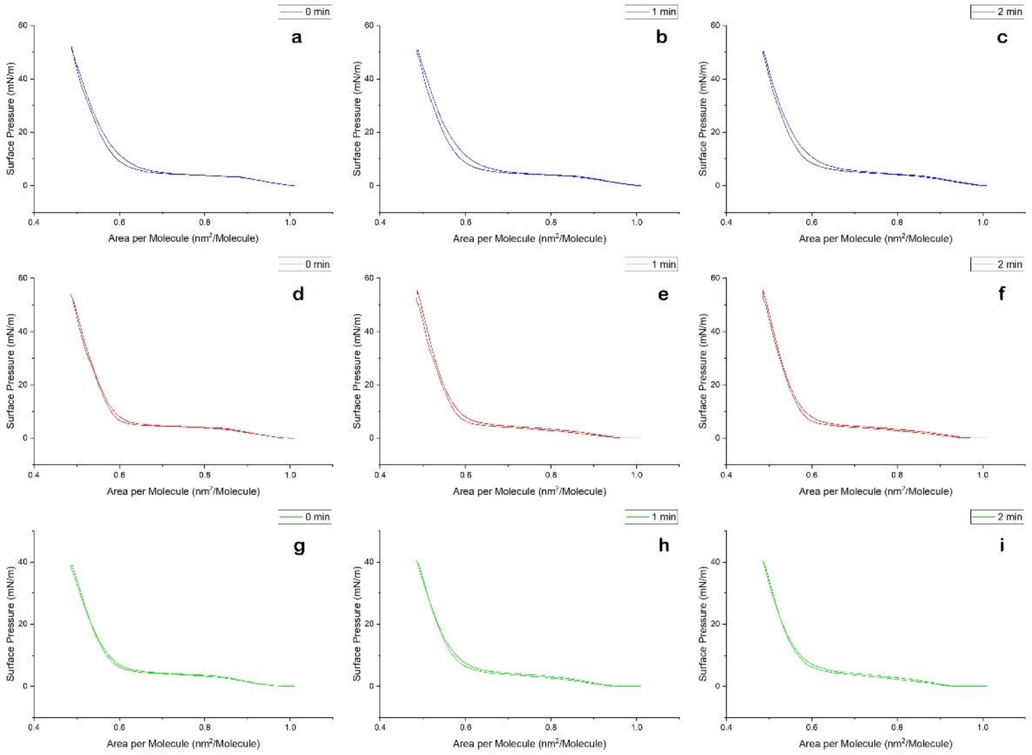

Figure S2. The isocycle curves of DPPC monolayer on (blue a-c) PBS 1X, (red d-f) water, and (green g-i) glucose 10 mM. The experiment was conducted at 20˚C. The residence times were selected for 0, 1, and 2 min shown in graphic legend.
